# Transcripts of antibacterial peptides in chicken erythrocytes infected with Marek’s disease virus

**DOI:** 10.1186/s12917-018-1678-7

**Published:** 2018-11-21

**Authors:** Sheng Niu, Ali Raza Jahejo, Fa-jie Jia, Xin Li, Guan-bao Ning, Ding Zhang, Hai-li Ma, Wei-fang Hao, Wen-wei Gao, Yu-jun Zhao, Shi-min Gao, Gui-lan Li, Jian-hui Li, Fang Yan, Rong-kun Gao, Yu-hai Bi, Ling-xia Han, George F. Gao, Wen-xia Tian

**Affiliations:** 1 College of Animal Science and Veterinary Medicine, Shanxi Agricultural University, Taigu, 030801 China; 2 Taiyuan Center for Disease Control and Prevention, Taiyuan, 030024 China; 3 CAS Key Laboratory of Pathogenic Microbiology and Immunology, Collaborative Innovation Center for Diagnosis and Treatment of Infectious Disease, Institute of Microbiology, Center for Influenza Research and Early-warning (CASCIRE), Chinese Academy of Sciences, Beijing, 100101 China; 4 Department of Laboratory Animal and Comparative Medicine, State Key Laboratory of Veterinary Biotechnology, Harbin Veterinary Research Institute, Chinese Academy of Agricultural Sciences, Harbin, 150001 China

**Keywords:** Chicken, Erythrocytes, *AvBDs*, *TLRs*, MDV

## Abstract

**Background:**

Chicken erythrocytes are involved in immunity through binding of toll-like receptors (TLRs) with their ligands to activate downstream signaling and lead to cytokine production in erythrocytes. Some avian β-defensins (*AvBDs*) are constitutively expressed in tissues and some others can be induced by various bacteria and viruses. However, the expression of *AvBDs* in erythrocytes has not yet been studied extensively.

**Results:**

The transcripts of eight *AvBDs* (*AvBD*1 to *AvBD*7, and *AvBD*9) and liver-expressed antimicrobial peptide-2 (*LEAP-*2) were found in normal chicken erythrocytes. The expression levels of *AvBD*2, 4 and 7 were significantly increased (*P* < 0.01), whereas the levels of *AvBD*1, 6 and 9 were significantly decreased (*P* < 0.01) after Marek’s disease virus (MDV) infection. The mRNA expression level of *LEAP-*2 was not significantly changed after MDV infection. Highest viral nucleic acid (VNA) of MDV in the feather tips among the tested time points was found at 14 days post-infection (d.p.i.). In addition, 35 MD5-related gene segments were detected in the erythrocytes at 14 d.p.i. by transcriptome sequencing.

**Conclusions:**

These results suggest that the *AvBDs* in chicken erythrocytes may participate in MDV-induced host immune responses.

## Background

Erythrocytes are the most abundant cells in circulation and the most important cells in the transportation and exchange of gases. In addition, several studies have reported that chicken erythrocytes play an important role in immunology [1]. For example, erythrocytes of vertebrates are nucleated and contain organelles in their cytoplasm, except in mammals [2]. It has been reported that the expression of cytokine transcripts in salmon erythrocytes were induced by infection with infectious salmon anaemia virus (ISAV). Similarly, cytokines, produced by trout erythrocytes when exposed to *Candida albicans*, enhanced the phagocytic capabilities [3, 4]. Moreover, the transcripts for many toll-like receptors (*TLRs*) were constitutively expressed in erythrocytes [1]. Chicken erythrocytes can induce the expression of transcripts for some cytokines and secrete thermo-labile molecules, which can enhance the antiviral capabilities of macrophages in response to *TLR* ligand treatments, such as type I interferons (*IFNs*) and interleukin (*IL*)-8 [1, 5].

Marek’s disease (MD) is a contagious lymphoproliferative disease caused by Marek’s disease virus (MDV), and can lead to tumors, lymphocyte proliferation, and ultimately death in chickens [6]. MDV has been evolving worldwide over the past few decades. The emerging MDV strains have changed from mild MDV (mMDV) to very virulent MDV (vvMDV) and very virulent plus MDV (vv + MDV) [7]. Host defense peptides (HDPs), also known as antimicrobial peptides, are a group of cationic amphipathic peptides that are produced by the host itself; they play a vital role in innate immunity and could kill various pathogenic microorganisms such as bacteria, fungi and even enveloped viruses [8–11]. Avian β-defensins (*AvBDs*) is the only type of defensin found in chickens [12–14]. Researchers are more interested in HDPs or their derivatives, because they believe HDPs could fight and protect against infectious diseases [15].

Recent studies have determined that *TLRs*, *AvBDs* and cytokines are involved in the response of the immune system in various tissues [16]. But the changes of expression level of *AvBDs* in chicken erythrocytes after MDV infection has not yet been investigated. The aim of the present study was to explore the transcriptional levels of *AvBDs* and liver-expressed antimicrobial peptide-2 (*LEAP-*2) in erythrocytes of chickens infected by MDV.

## Results

### *AvBDs* and *LEAP*-2 genes expression detected in chicken erythrocytes

**Fig. 1:**
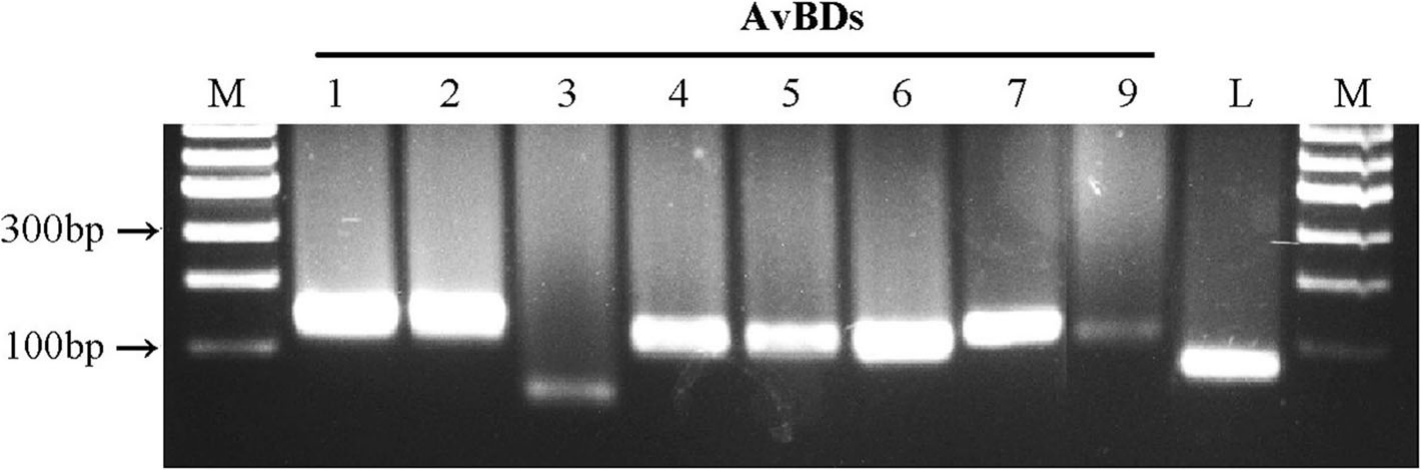
Expression pattern of *AvBDs* and *LEAP*-2 genes in the erythrocytes of 3-week-old roosters. All of the *AvBDs* and *LEAP*-2 were detected by RT-PCR. M = size marker, L = *LEAP*-2

The results of the reverse transcription-polymerase chain reaction (RT-PCR) showed that the genes expression of *AvBD*1 to *AvBD*7, *AvBD*9, and *LEAP-*2 was detected in the normal commercial chicken erythrocytes, but not for *AvBD*8, and *AvBD*10 to *AvBD*14 (Fig. 1), and no bands were found in the negative controls. *AvBD*1 to *AvBD*7, *AvBD*9 and *LEAP-*2 genes expression was also detected in specific pathogen-free (SPF) chicken erythrocytes (data not shown). These detected genes expression was further confirmed by Sanger sequencing and Real-time PCR.

### Virus load in the feather tips of MDV-infected chickens

**Fig. 2:**
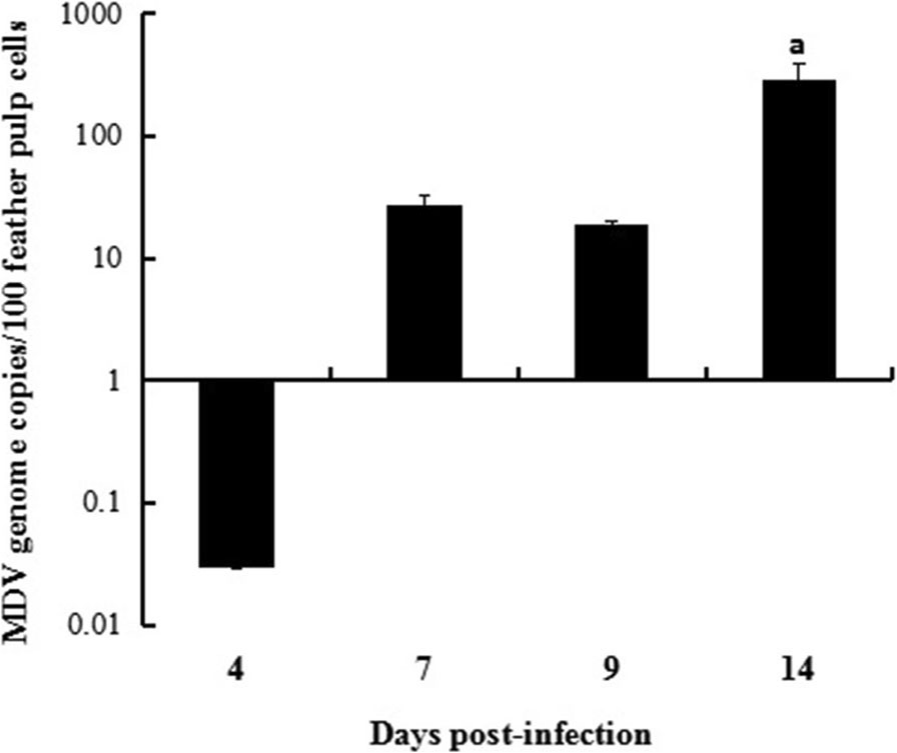
MDV load in feather tips on 4, 7, 9 and 14 d.p.i. (*n* = 3). Chickens were infected with MDV. Feather tips were obtained on 4, 7, 9 and 14 d.p.i., and the virus loads were measured by duplex real-time PCR. The error bars represent standard error of the mean value. The data of virus load was subjected to one-way ANOVA and then analyzed by the Tukey’s pairwise comparison to identify treatment differences, a = significant when compared to MDV-infected chickens sampled on 4, 7 and 9 d.p.i. (*P* < 0.05)

No severe clinical symptom except late phase depression was observed in 14 d experimental period. The copy numbers of MDV were tested at 4, 7, 9 and 14 days post-infection (d.p.i.) by duplex real-time PCR (Fig. 2). The results showed that the MDV was detected after 7 d.p.i., and the MDV copy number at 14 d.p.i. was significantly higher than that of all other time points. MDV nucleic acid was not detected in the control group.

### Detection of MDV-related genes in erythrocytes

To determine whether MDV invaded chicken erythrocytes which could activate *AvBDs* expression against the infection, we performed a search for virus genomes in the transcriptome of erythrocytes on 14 d.p.i. (SRA accession No: SRP136403) when the MDV load was significantly higher than that of all other time points. The results showed that the transcripts of 35 MD5-related genes were detected (Table 1). These genes were mainly phosphoprotein pp24, major capsid protein, assembly protein, major DNA-binding protein, deneddylase, DNA polymerase processivity factor, tegument protein VP16, and deoxyuridine triphosphatase, in which the copy number of phosphoprotein pp24 that act as an integral component of membrane was the most abundant. These results indicated that erythrocytes had been invaded by MDV.

**Table 1:** The identities of 35 MD5-related gene transcripts detected in chicken erythrocytes after MDV infection

ID	Length(nt)	Count (Reads numbers)	Swissprot_annotation (strain Chicken/Md5/ATCC VR-987)	nr_annotation [Gallid herpesvirus 2]
MDV008	2608	17	Phosphoprotein pp24	protein pp24
MDV018	2169	1	Portal protein UL6	capsid portal protein
MDV021	2526	1	Replication origin-binding protein	DNA replication origin-binding helicase
MDV023	3786	5	Tegument protein UL11	myristylated tegument protein
MDV030	960	1	Triplex capsid protein VP23	capsid triplex subunit 2
MDV031	4182	8	Major capsid protein	HSV-1 UL19-like protein
MDV034	2442	1	Envelope glycoprotein H	envelope glycoprotein H
MDV038	1992	6	Assembly protein	capsid maturation protease
MDV040	2598	1	Envelope glycoprotein B	envelope glycoprotein B
MDV042	3576	6	Major DNA-binding protein	single-stranded DNA-binding protein
MDV043	3663	3	DNA polymerase catalytic subunit	DNA polymerase catalytic subunit
MDV044	903	1	Virion egress protein UL31	nuclear egress lamina protein
MDV046	1926	1	Packaging protein UL32	DNA packaging protein UL32
MDV047	834	1	Virion egress protein UL34	nuclear egress membrane protein
MDV049	10,029	7	Deneddylase	large tegument protein
MDV050	3141	1	Capsid assembly protein UL37	tegument protein UL37
MDV051	1413	1	Triplex capsid protein	capsid triplex subunit 1
MDV052	2469	2	Ribonucleoside-diphosphate reductase large subunit	ribonucleotide reductase subunit 1
MDV053	1032	1	Ribonucleoside-diphosphate reductase small chain	ribonucleotide reductase subunit 2
MDV055	1506	6	DNA polymerase processivity factor	DNA polymerase processivity subunit
MDV059	1707	4	Tegument protein UL46	tegument protein VP11/12
MDV060	2427	3	Tegument protein UL47	tegument protein VP13/14
MDV061	1284	6	Tegument protein VP16	transactivating tegument protein VP16
MDV062	750	1	Tegument protein VP22	tegument protein VP22
MDV063	1311	9	Deoxyuridine 5&apos; −triphosphate nucleotidohydrolase	deoxyuridine triphosphatase
MDV065	750	1	Tegument protein UL51	tegument protein UL51
MDV068	1422	3	mRNA export factor ICP27	multifunctional expression regulator
MDV069	810	1	Uncharacterized gene 69 protein	protein LORF4
MDV070	501	1	Tegument protein UL55	nuclear protein UL55
MDV071	585	1	Uncharacterized gene 71 protein	myristylated tegument protein CIRC
MDV072	2712	5	Uncharacterized gene 72 protein	protein LORF5
MDV072.5	873	3	–	membrane protein UL56
MDV088	540	3	Transcriptional regulator ICP22	regulatory protein ICP22
MDV089	642	2	Virion protein US10	virion protein US10
MDV094	4716	3	Envelope glycoprotein D	Envelope glycoprotein D

### Quantitative PCR analysis for *AvBDs* and *LEAP-2* expression in the chicken erythrocytes in response to MDV stimulation

**Fig. 3:**
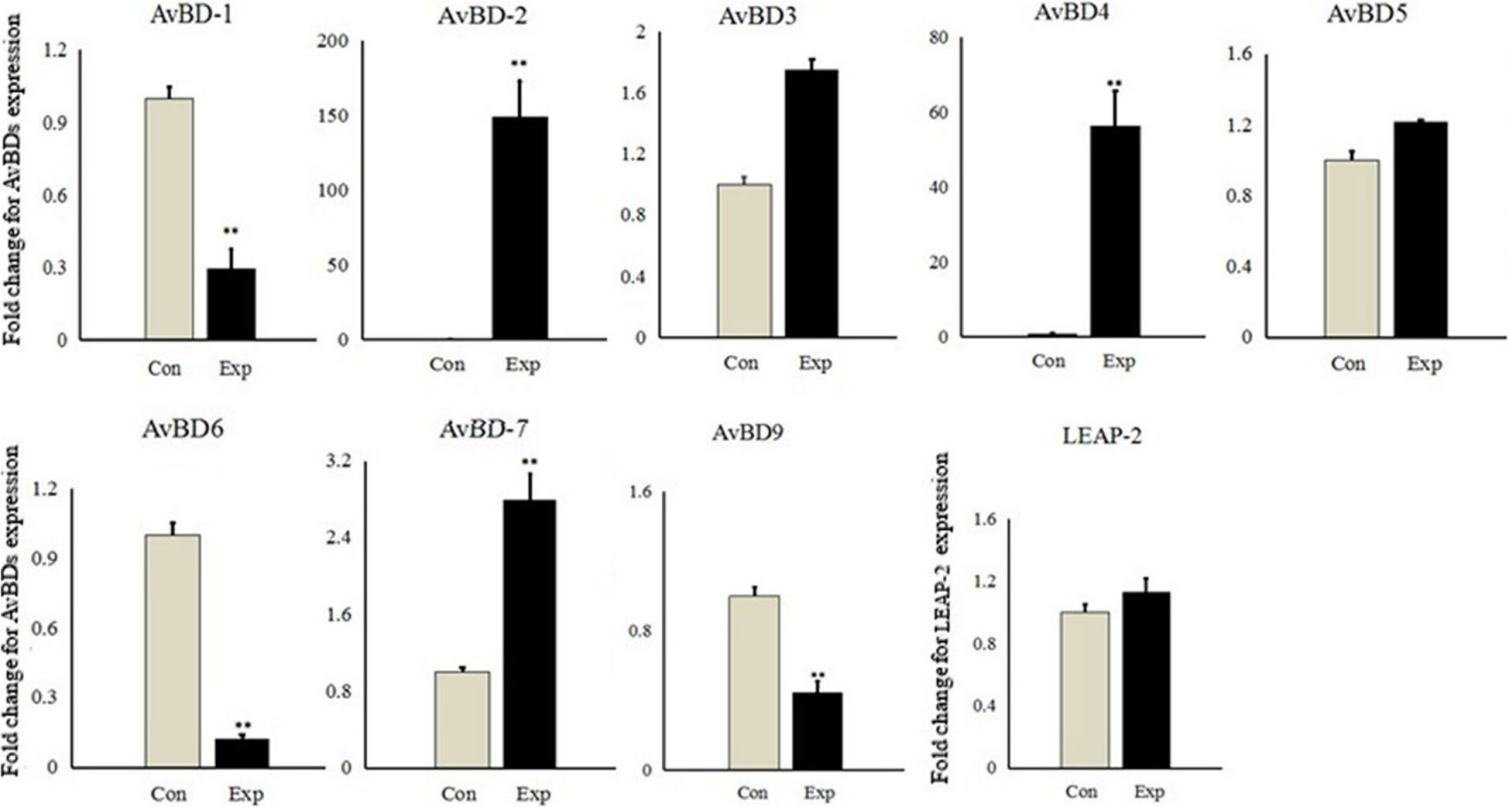
The expression pattern of mRNAs of *AvBDs* in chicken erythrocytes on 14 d.p.i. The experimental groups of this study were as follows: the control group (Con, PBS-injection, *n* = 3) and the infection group (Exp, MDV-injection, n = 3). Expression levels of *AvBDs* were calculated relative to that of the housekeeping gene *18S rRNA* by using quantitative real-time PCR. The data represent mean fold changes from PBS-treated controls ± standard error. Statistical significance between the infection group and the control group was analyzed using t-test. *P < 0.05, **P < 0.01

To further investigate the functions of these genes during MDV infections, their expression levels in the erythrocytes were analyzed. We utilized real-time RT-PCR to quantify the repertoire of *AvBDs* expressed in chicken erythrocytes in response to MDV on 14 d.p.i.(Fig. 3). The expression levels of *AvBD*2, 4 and 7 were significantly upregulated compared to the control group (*P* < 0.01). However, the gene transcript levels of *AvBD*1, 6 and 9 in the experimental group were significantly lower than those in the control group (*P* < 0.01). There was no significant difference for *AvBD*3, *AvBD*5 and *LEAP-*2 between the two groups.

## Discussion

Recent studies have reported that erythrocytes play an important role in immune responses. It has been found that trout and chicken erythrocytes can detect and specifically respond to different pathogen-associated molecular patterns (PAMPs) [5]. In the present study, it was observed that *AvBDs* and *LEAP-*2 transcripts are expressed not only in tissues but also in erythrocytes. This result provides a new perspective for poultry health, as the expression of these transcripts may be linked to the protection of chickens from pathogens. Primary literatures showed that *AvBDs* are not only largely associated to reproductive organs but also to bone marrow [17–19], and more generally to mucosal tissues. The transcripts for 13 types of *AvBDs* were expressed in chicken reproductive organs [20]. It has been reported that *AvBD*1, 2 and *AvBD*4 to *AvBD*7 were highly expressed in bone marrow of chicken [18, 19, 21]. Strong expression of *AvBDs* transcripts was found throughout the digestive tract such as in the esophagus, craw, proventriculus, and intestinal tissues, except for *AvBD*11 [16, 22, 23]. This is similar to our results that *AvBD*1 to *AvBD*7 and *AvBD*9 transcripts were detected in chicken erythrocytes. Moreover, many *AvBDs* also were found and regulated by both pathogenic and attenuated infectious bronchitis virus (IBV) strains in many highly vascularized tissues, such as liver, spleen and kidney [24, 25]. The expression of *AvBDs* was also observed in other tissues such as the respiratory tract and skin [12, 17, 21]. As the most abundant circulating cells, erythrocytes are distributed in all of these tissues. All of the above support the hypothesis that erythrocytes can participate in the immune response in various tissues, especially highly vascularized tissues, by releasing *AvBDs* to fight pathogens.

The results from current study indicated that MDV genome load in the feather tips was significantly increased at 14 d.p.i.. Similar results were reported by Abdul-Careem et al [26]. This could be because some latent MDV was reactivated and started the second cytolytic phase by 14 d.p.i. [6]. The transcripts of 35 MD5-related genes in erythrocytes indicated that MDV invaded the membrane or intracellular of erythrocytes. The explanation for the results could be 1) the viral PAMPs were recognized by the pattern recognition receptors (PRRs) on the surface of erythrocytes; 2) MDV invaded and replicated in the erythrocytes; 3) there was an interaction between MDV and erythrocytes. However, the molecular mechanism needs to be further elucidated.

*TLRs* are associated with immune cells and other tissue cells [17]. It was recently found that chicken erythrocytes constitutively express transcripts for many *TLRs* as well as for some cytokines and other immune-related genes such as *IFN-α*, *IFN-β*, and *IL-8* [1]. *AvBDs* can be induced by the interaction of *TLRs* with their ligands and proinflammatory cytokines [27, 28]. The results of the present study show that it is possible that erythrocytes can participate in some of these aspects. Furthermore, many MDV-related genes and differentially expressed *AvBD*2 were detected in the transcriptome of erythrocytes, suggesting the expression of *AvBDs* in erythrocytes may participate in the antiviral response against MDV infection. The significant upregulation of *AvBD*2, 4 and 7 was found after MDV infection, which may result from the activation of broader innate immune signaling pathways after PRRs-viral PAMPs interaction, and this still needs further researches to confirm. We found that the upregulated expression of *AvBD*2 is the most significant result obtained after MDV infection. Similarly, some studies found that *AvBD*2 was also upregulated by the infections of pathogenic IBV and pigeon paramyxovirus type 1 (PPMV-1) in most tissues [24]. Furthermore, it has been reported that *AvBD*2 is the major defensin in heterophiles [27, 29]. We speculate that *AvBD*2 may be expressed as functional proteins. In duck tissues, significant antiviral activities of three *AvBDs* (Apl_*AvBD*4, 7 and 12) were shown against duck hepatitis virus (DHV) [30]. In another study, the recombinant *AvBD*2, 6 and 12 displayed the obvious anti-viral activity against IBV in vitro [24]. These suggested that *AvBD*2, 4, 6 and 7 may have strong antiviral activities in chicken erythrocytes. Moreover, we also found that the transcripts of *AvBD*1, 6 and 9 were significantly downregulated after MDV infection; this may be associated to the viral immunomodulators that fight against these *AvBDs* after MDV infection. The *LEAP-*2 expression in erythrocytes during MDV infection has also been found; thus, we speculate that *LEAP-*2 might also be an effector of innate immune signaling as a response to some pathogens [31].

The modulation of *AvBDs* in response to the bacterial components or ligands for *TLRs* in chickens have already been studied [1, 20, 23], but the reaction of *AvBDs* in response to MDV in chicken erythrocytes has not yet been investigated. Based on the expressed repertoire of *AvBDs*, it is possible that erythrocytes can respond both virus and bacteria, which is supported by the fact that *AvBD*2 which is highly overexpressed in response to MDV possess a strong antibacterial activity [19]. This finding is also supported by another fact that the transcript of type I *IFNs* are also expressed in chicken erythrocytes and are upregulated after the treatment with poly I:C or CpG ODN [1], such that a second wave of signaling pathways would be activated to synthesize IFN-regulated proteins contributing to the antiviral response and inhibiting virus replication [32]. Another study showed the expression of several *AvBDs* was closely correlated to the expression of *TLR*3, *TLR*7 and *TLR*15 in tissues [33]. All the above clues supported the possible defensive mechanism of chicken erythrocytes that the PAMPs could be recognized by various PRRs, such as *TLRs* and scavenger receptor (*SR*). Cytokines, *AvBDs* and other molecules will be activated and modulate host immune response when pathogens invade into the blood.

## Conclusions

In conclusion, the transcripts of eight *AvBDs* and *LEAP-*2 were identified in chicken erythrocytes. The transcripts of 35 MD5-related genes were found in erythrocytes after MDV infection. MDV induced an immune response wherein *AvBD*2, 4 and 7 were significantly upregulated and *AvBD*1, 6 and 9 were significantly downregulated. Further studies are needed on the mechanism of *AvBDs* in the response of chicken erythrocytes to MDV infections, which could help to prevent and control MDV in the future.

## Methods

### Experiment 1. Detection of *AvBDs* and *LEAP*-2 genes expression in chicken erythrocytes

#### Experimental animals

Three 3-week-old roosters (Hy-Line Brown) were purchased from a commercial supplier in Taigu County. The roosters were kept in cages, under an artificial light regimen of 14 h day-time/10 h night-time. A diet appropriate for their age was provided ad libitum. Before exsanguination and necropsy, injection of pentobarbital was used along with the standard protocols of euthanasia to minimize animals suffering.

#### Erythrocyte collection

Venous blood was drawn from these chickens (2 mL per bird) and mixed with the same volume of Alsever’s solution (Solarbio, Beijing, China). The diluted blood was added carefully into a 4 mL Histopaque-1119 solution (Sigma–Aldrich, Oakville, ON) and centrifuged at 500 *g* for 20 min; subsequently, the supernatant containing platelets and leukocytes-was removed. Subsequent procedures were performed as described earlier [34]. Isolated erythrocytes were determined to be > 99.9% pure by Wright–Giemsa staining.

#### Extraction of total RNA from chicken erythrocytes, and cDNA synthesis

Total RNA was purified from erythrocytes isolated from blood samples using RNAiso Plus (Takara Bio Inc., Dalian, China) according to the manufacturer instructions, and dissolved in 20 μL RNase-free water. Purified RNAs were examined by 1.5% agarose gel electrophoresis and a NanoDrop Bioanalyzer ND1000 (Labtech, Uckfield, UK). The criteria for assessing the quality of RNA were that *28S* and *18S rRNA* could be seen in the gel and the A260/A280 ratio was between 1.9–2.1. cDNA was synthesized from 500 ng total RNAs using the PrimeScript RT reagent Kit with gDNA Eraser (Takara Bio Inc., Dalian, China) according to manufacturer instructions. The cDNA samples were diluted at 1:10 in sterile water and stored at − 20 °C.

#### Primer design and PCR

Primers were designed by Primer Express 3.0 (Applied Biosystems, Foster City, CA, USA) and synthesized by Shanghai Generay Biotech Co., Ltd. (Shanghai, China). GenBank accession numbers of the reference sequences used for primer design are presented in Table 2. The PCR was conducted in a volume of 20 μL of reaction mixture containing 2 μL of cDNA, 0.4 μL at 10 pmol/μL of each of forward and reverse primers, 10 μL 2 × MasterMix (Real-Times Biotechnology Co., Ltd., Beijing, China) and 7.2 μL double-distilled H_2_O. The cDNA was replaced by water as negative control for each gene. Amplification for *AvBDs* or *LEAP-*2 was performed in a Gradient Thermocycler (Eppendorf AG, Germany). The optimum thermal cycling procedure was as follows: 95 °C for 3 min; 35 cycles of 95 °C for 30 s, 55 to 57 °C (the optimal temperature of *AvBDs*, listed in Table 2) for 30 s, and 72 °C for 1 min, followed by 72 °C for 5 min. The PCR results were detected by electrophoresis in 1.5% TAE agarose gels, and imaged by GelDoc-It Imaging Systems (Ultra-Violet Products, USA).

**Table 2:** Primer sequences and reference genes used for PCR

Target Genes	Primer sequence(5′ → 3′)	Annealing temperature	Sizes(bp)	GenBank Accession number of the reference gene
*AvBD*1	F: GGCTCTAGGAAGGAAGTCA R: GCAGCAGAGGTAAAATCTTG	57 °C	112	NM_204993.1
*AvBD*2	F: GCACTCCAGGTTTCTCCA R: GGCGTCCGACTTTGATTA	57 °C	110	DQ677633.1
*AvBD*3	F: AGAGGAGGATTCTGTCGTGTT R: CAACCTCATATGCTCTTCCA	55 °C	100	NM_204650.2
*AvBD*4	F: TCATGGAGCTGTGGGCTTTT R: AGCATTCCCATAAGGGCATT	55 °C	100	NM_001001610.2
*AvBD*5	F: ATTACCCCAGGACTGTGA R: GCAGCAGAAGTCTTCCTT	55 °C	100	NM_001001608.2
*AvBD*6	F: CTGCTGCTGTCTGTCCTCTT R: TGCAGACACCCCTTTGATAT	55 °C	100	NM_001001193.1
*AvBD*7	F: CTATTGATACTTGTTGGCTTCG R: AACTCCTCCATCCCCTTG	57 °C	122	AY621322.1
*AvBD*9	F: CCAGCTTACAGCCAAGAAGA R: TCCCAATGTCAACTGAAGGA	55 °C	100	NM_001001611.2
*LEAP*-2	F: ACTCTGGAATTCTGCCTGATGACA R: CATCTGCATCCGTGCCTGA	57 °C	66	NM_001001606.1
*18S rRNA*	F: TTCCGATAACGAACGACAC R: GACATCTAAGGGCATCACAG	55 °C	139	FM165414

### Experiment 2. *AvBDs* and *LEAP*-2 gene expression and virus load in erythrocytes and feather tips of MDV-infected chickens

#### Virus and animals

Twenty 1-day-old SPF chickens (B15 haplotype), very virulent MDV strain MD5, and recombinant plasmid pMD-O containing the *ovotransferrin* gene and recombinant plasmid pMD-M containing the MDV Eco Q (*meq*) gene were provided by Dr. Lingxia Han of Harbin Veterinary Research Institute, Chinese Academy of Agriculture Sciences (Harbin, China). The strain of MDV (MD5) was preserved in liquid nitrogen until use.

#### Animal infection

The chickens were equally divided into two groups: an experimental group and a control group. The animal experiment was carried out in an Animal Biosafety Level 2 Laboratory. The MDV was diluted properly with sterile phosphate buffered saline (PBS) (Solarbio, Beijing, China). Each chicken in the experimental group was intra-abdominally infected with MDV at a dose of 500 plaque-forming units (PFU)/500 μL. The control group was injected with the same volume of PBS. On 4, 7, 9 and 14 d.p.i., 4–5 feather tips were collected from each bird in the experimental and control groups and preserved in liquid nitrogen immediately. Anticoagulated blood samples were collected at 14 d.p.i. for both groups and used for total RNA extraction as described in Experiment 1. Before exsanguination and necropsy, injection of pentobarbital was used along with the standard protocols of euthanasia to minimize animals suffering. All these tests were performed by Lingxia Han at Harbin Veterinary Research Institute, Chinese Academy of Agriculture Sciences (Harbin, China).

#### Library preparation and sequencing

RNA was extracted from the erythrocytes. A total of 1 μg purified RNA per sample was used for next-generation sequencing (NGS). Sequencing libraries were generated using NEBNext®Ultra™ RNA Library Prep Kit for Illumina® (NEB, USA) following the manufacturer’s instruction, and index codes were added to attribute sequences to each sample as described previously [35]. The libraries were sequenced by Illumina HiSeq 2500.

#### Transcriptome analysis

To identify the viral sequence, the clean reads were mapped to the reference genomic sequence by Tophat2 tools software [36], and each unigene was then transformed into FPKM (Fragments Per Kilo base per Million mapped reads) by using RSEM software [37]. Annotations of the unigenes were searched using BLAST against the Nr (non-redundant) and the Swiss-Prot databases (E-value ≤10^− 5^).

#### Detection of AvBDs and LEAP-2 mRNA expression in erythrocytes

RNA was extracted from the erythrocytes on 14 d.p.i.. The quantitative expression analysis of *AvBDs* and *LEAP-*2 genes were performed with real-time RT-PCR using a TaKaRa SYBR Premix Ex Taq™II (Takara Bio Inc.). The total volume of real-time PCR mixture contained 6 μL 1× SYBR Premix Ex TaqII, 0.1 μL ROX dye II, 1.5 pmol each of forward and reverse primers, 1 μL of diluted cDNA, and 2.6 μL of double distilled H_2_O. The reaction mixture was placed into MicroAmp® Optical 8-Cap Strip (Applied Biosystems), and real-time PCR was performed following user instructions of the QuantStudio™ 6 (Applied Biosystems). Briefly, each reaction involved a pre-incubation at 95 °C for 3 min, followed by 42 cycles of 95 °C for 30 s, 55 °C - 57 °C (T_A_ as per primer) for 30 s, and extension at 72 °C for 10 s. The expression level of *AvBDs* and *LEAP-*2 were calculated relative to that of the housekeeping gene *18S rRNA* using the QuantStudio™ 6 Flex Real-Time PCR System Software (Applied Biosystems, USA).

#### DNA extraction from feather tips

DNA was extracted from feather tips collected at 4, 7, 9 and 14 d.p.i. using Trizol reagent (Invitrogen, Carlsbad, California, USA) as described previously [38]. The purity and concentration of purified DNAs were detected by a NanoDrop Bioanalyzer ND1000 (Labtech). The feather tips DNA was then diluted to 100 ng/μL for future use.

#### Primers and probes for meq and ovotransferrin genes

Primers and probes of the duplex real-time PCR (Table 3) for MDV *meq* gene and *ovotransferrin* gene followed that by Islam et al. [39] and Baigent et al. [40], respectively. Chicken *ovotransferrin* genomes gene was used as DNA extraction reference.

**Table 3:** Sequences of the primers, probes and reference genes used for the duplex real-time PCR assay

Primer/probe name	Primer/probe sequence(5′ → 3′)	Target gene	GenBank Accession number
meq-F	GGAGCCGGAGAGGCTTTATG		
meq-R	ATCTGGCCCGAATACAAGGAA	MDV Eco Q	M89471
meq-P	(FAM)CGTCTTACCGAGGATCCCGAACAGG(TAMRA)		
ovo-F	CACTGCCACTGGGCTCTGT		
ovo-R	GCAATGGCAATAAACCTCCAA	ovotransfenrin	Y00407
ovo-P	(ROX)AGTCTGGAGAAGTCTGTGCAGCCTCCA(BHQ2)		

FAM: Carboxy fluorescein, TAMRA: Carboxy tetramethyl rhodamine, ROX: Carboxy-X-rhodamine, BHQ2: Black Hole Quencher 2

#### Quantification of virus loads by real-time PCR

Serial 10-fold dilutions (10^4^–10^10^ copies/μL) of the recombinant plasmids of pMD-O and pMD-M were used to generate a standard curve for *ovotransferrin* and *meq* gene. The duplex real-time PCR was performed in a 25-μL volume. The reaction mixture included 2.5 μL 10 × Ex Taq buffer; 1 μL dNTPs; 0.2 μL HS Ex Taq; 8 pM *meq*-P and *ovo*-P probes; 10 pM *meq*-F, *meq*-R, *ovo*-F and *ovo*-R primers; 1 μL pMD-O and pMD-M. Sterilized H_2_O was added to bring the final volume to 25 μL. The protocol for the reaction was as follows: 95 °C for 5 min; 40 cycles of 94 °C for 15 s, 59.5 °C for 50 s. The fluorescence intensity of the reporter dye (FAM and ROX) was measured during the 59.5 °C annealing/extension step. The data analysis was performed using an iCycler Thermal Cycler (BioRad, Hercules, CA, USA) generating the standard curve. For the detection of the copy numbers of MDV in feather tips on 4, 7, 9 and 14 d.p.i., the DNAs in feather tips were used as templates for the duplex real-time PCR assay to detect the *meq* and *ovotransferrin*. The copy numbers of MDV were calculated using the standard curve.

#### Statistical analysis

The 2^−ΔΔCt^ method was used to calculate the real-time PCR data. Data represent mean fold change from PBS-treated controls ± standard error. Statistical analyses were performed using SPSS statistical software 17.0 (IBM Company, New York, NY, USA). The data of virus load was subjected to one-way ANOVA and then analyzed by the Tukey’s pairwise comparison to identify treatment differences which were considered significant when *P* < 0.05. Statistical significance of each gene expression between the experimental group and the control group was analyzed using t-test for independent samples. Differences were considered significant when the *P*-value was *P < 0.05 or **P < 0.01.

## Abbreviations

*AvBD*s: Avian β-defensins
HDPs: Host defense peptides
IBV: Infectious bronchitis virus
*IFNs*: Type I interferons
*IL*: Interleukin
ISAV: Infectious salmon anaemia virus
*LEAP*-2: Liver-expressed antimicrobial peptide-2
MD: Marek’s disease
MDV: Marek’s disease virus
*meq*: MDV Eco Q
mMDV: Mild MDV
NGS: Next-generation sequencing
PAMPs: Pathogen associated molecular patterns
PBS: Phosphate buffered saline
PFU: Plaque-forming units
PPMV-1: Pigeon paramyxovirus type 1
PRRs: Pattern recognition receptors
RT-PCR: Reverse transcription polymerase chain reaction
SPF: Specific pathogen-free
*SR*: Scavenger receptor
*TLRs*: Toll-like receptors
VNA: Viral nucleic acid
vvMDV: Very virulent MDV
